# To admit or not to admit? The suitability of critical care admission criteria

**DOI:** 10.1186/cc11118

**Published:** 2012-03-20

**Authors:** D Marriott, Z Turner, N Robin, S Singh

**Affiliations:** 1 Countess of Chester Hospital, Chester, UK

## Introduction

During the 2010/2011 winter the H1N1 influenza pandemic placed increased demand on critical care services, prompting our department to devise a modified triage tool for the ICU to be implemented at a time of exceptional bed crisis [1]. Scoring systems such as APACHE or Sequential Organ Failure Assessment (SOFA) have been used to predict mortality and optimize critical care service utilization [2]. This audit aimed to validate our triage tool for patients admitted to the ICU.

## Methods

We retrospectively examined patient notes for all admissions to our adult ICU during December 2010 and January 2011. Patient admission criteria (SpO_2 _<90% on FiO_2 _>85%, respiratory acidosis pH ≤7.2, respiratory failure or airway compromise, systolic pressure <90 mmHg, SOFA score ≥7) or refusal criteria (SOFA score ≥12, severe trauma, unwitnessed or non-VF arrest, severe life-limiting condition) were recorded with outcome data.

## Results

We analysed 27 sets of notes. Twenty-two patients (81%) fulfilled at least one admission and no refusal criteria. Two patients (7%) had documented refusal criteria. The first of these had a severe life-limiting condition, staying 29 days in the ICU and a further 65 days in hospital. The second was admitted post non-VF arrest, dying after 2 days in the ICU. Three patients (11%) met no admission criteria. These patients stayed between 4 and 6 days in critical care with total hospital stays of 18 to 98 days, one requiring 30 days of rehabilitation.

## Conclusion

The proposed admission criteria concurred with clinical decision-making in 81% of admissions. The patients that met refusal criteria required either prolonged hospital stay or had short survival times and may not represent optimal utilization of critical care facilities during a time of increased demand. Those patients not meeting the admission criteria had short critical care stays illustrating that rigid admission requirements may exclude patients who could benefit from critical care. A standardized set of admission criteria may supplement decision-making during times of increased critical care demand and strengthen documentation of those decisions. However, no set of criteria can replace clinical judgement in critical care admission.
